# Supplementary material for: The transcriptional response of cortical neurons to concussion reveals divergent fates after injury
Source: bioRxiv. 2024 Mar 11:2024.02.26.581939. Originally published 2024 Feb 28. Preprint. [Version 2] doi: 10.1101/2024.02.26.581939 (PMC10925231; doi:10.1101/2024.02.26.581939)
Supplement: Supplementary file 2 [file media-1.pdf]

Cell Type	Count
L2/3 IT CTX-1	943
L2/3 IT CTX-2	173
L4/5 IT CTX	173
L5 IT CTX	35
L5 NP CTX	7
L5 PT CTX	647
L6 CT CTX	85
L6 IT CTX	223
L6b CTX	1
Lamp5	27
Pvalb	268
Sncg	11
Sst	318
Vip	64
